# *In vitro* comparison of human plasma-based and self-assembled tissue-engineered skin substitutes: two different manufacturing processes for the treatment of deep and difficult to heal injuries

**DOI:** 10.1093/burnst/tkad043

**Published:** 2023-10-31

**Authors:** Álvaro Sierra-Sánchez, Brice Magne, Etienne Savard, Christian Martel, Karel Ferland, Martin A Barbier, Anabelle Demers, Danielle Larouche, Salvador Arias-Santiago, Lucie Germain

**Affiliations:** LOEX Tissue Engineering Laboratory and Department of Surgery, Faculty of Medicine, Université Laval, 1401 18e rue, Québec (Québec) G1J 1Z4, Canada; CHU de Québec – Université Laval Research Center, Division of Regenerative Medicine, 1401 18e rue, Québec (Québec) G1J 1Z4, Canada; Unidad de Producción Celular e Ingeniería Tisular (UPCIT), Virgen de las Nieves University Hospital, ibs. GRANADA, Andalusian Network for the design and translation of Advanced Therapies, Av. de las Fuerzas Armadas, Nº2, 4ª Planta Ed. de Gobierno, 18014, Granada, Spain; LOEX Tissue Engineering Laboratory and Department of Surgery, Faculty of Medicine, Université Laval, 1401 18e rue, Québec (Québec) G1J 1Z4, Canada; CHU de Québec – Université Laval Research Center, Division of Regenerative Medicine, 1401 18e rue, Québec (Québec) G1J 1Z4, Canada; LOEX Tissue Engineering Laboratory and Department of Surgery, Faculty of Medicine, Université Laval, 1401 18e rue, Québec (Québec) G1J 1Z4, Canada; CHU de Québec – Université Laval Research Center, Division of Regenerative Medicine, 1401 18e rue, Québec (Québec) G1J 1Z4, Canada; LOEX Tissue Engineering Laboratory and Department of Surgery, Faculty of Medicine, Université Laval, 1401 18e rue, Québec (Québec) G1J 1Z4, Canada; CHU de Québec – Université Laval Research Center, Division of Regenerative Medicine, 1401 18e rue, Québec (Québec) G1J 1Z4, Canada; LOEX Tissue Engineering Laboratory and Department of Surgery, Faculty of Medicine, Université Laval, 1401 18e rue, Québec (Québec) G1J 1Z4, Canada; CHU de Québec – Université Laval Research Center, Division of Regenerative Medicine, 1401 18e rue, Québec (Québec) G1J 1Z4, Canada; LOEX Tissue Engineering Laboratory and Department of Surgery, Faculty of Medicine, Université Laval, 1401 18e rue, Québec (Québec) G1J 1Z4, Canada; CHU de Québec – Université Laval Research Center, Division of Regenerative Medicine, 1401 18e rue, Québec (Québec) G1J 1Z4, Canada; LOEX Tissue Engineering Laboratory and Department of Surgery, Faculty of Medicine, Université Laval, 1401 18e rue, Québec (Québec) G1J 1Z4, Canada; CHU de Québec – Université Laval Research Center, Division of Regenerative Medicine, 1401 18e rue, Québec (Québec) G1J 1Z4, Canada; LOEX Tissue Engineering Laboratory and Department of Surgery, Faculty of Medicine, Université Laval, 1401 18e rue, Québec (Québec) G1J 1Z4, Canada; CHU de Québec – Université Laval Research Center, Division of Regenerative Medicine, 1401 18e rue, Québec (Québec) G1J 1Z4, Canada; Unidad de Producción Celular e Ingeniería Tisular (UPCIT), Virgen de las Nieves University Hospital, ibs. GRANADA, Andalusian Network for the design and translation of Advanced Therapies, Av. de las Fuerzas Armadas, Nº2, 4ª Planta Ed. de Gobierno, 18014, Granada, Spain; Department of Dermatology, Virgen de las Nieves University Hospital, Av. Madrid, Nº11–15, 18012, Granada, Spain; Department of Dermatology, Faculty of Medicine, University of Granada, Av. de la Investigación, Nº11, 18016, Granada, Spain; LOEX Tissue Engineering Laboratory and Department of Surgery, Faculty of Medicine, Université Laval, 1401 18e rue, Québec (Québec) G1J 1Z4, Canada; CHU de Québec – Université Laval Research Center, Division of Regenerative Medicine, 1401 18e rue, Québec (Québec) G1J 1Z4, Canada

**Keywords:** Biomaterial, Burn, Fibrin, Human plasma, Self-assembly, Skin substitute, Tissue engineering

## Abstract

**Background:**

The aim of this *in vitro* study was to compare side-by-side two models of human bilayered tissue-engineered skin substitutes (hbTESSs) designed for the treatment of severely burned patients. These are the scaffold-free self-assembled skin substitute (SASS) and the human plasma-based skin substitute (HPSS).

**Methods:**

Fibroblasts and keratinocytes from three humans were extracted from skin biopsies (N = 3) and cells from the same donor were used to produce both hbTESS models. For SASS manufacture, keratinocytes were seeded over three self-assembled dermal sheets comprising fibroblasts and the extracellular matrix they produced (n = 12), while for HPSS production, keratinocytes were cultured over hydrogels composed of fibroblasts embedded in either plasma as unique biomaterial (Fibrin), plasma combined with hyaluronic acid (Fibrin-HA) or plasma combined with collagen (Fibrin-Col) (n/biomaterial = 9). The production time was 46–55 days for SASSs and 32–39 days for HPSSs. Substitutes were characterized by histology, mechanical testing, PrestoBlue™-assay, immunofluorescence (Ki67, Keratin (K) 10, K15, K19, Loricrin, type IV collagen) and Western blot (type I and IV collagens).

**Results:**

The SASSs were more resistant to tensile forces (*p-*value < 0.01) but less elastic (*p-*value < 0.001) compared to HPSSs. A higher number of proliferative Ki67^+^ cells were found in SASSs although their metabolic activity was lower. After epidermal differentiation, no significant difference was observed in the expression of K10, K15, K19 and Loricrin. Overall, the production of type I and type IV collagens and the adhesive strength of the dermal-epidermal junction was higher in SASSs.

**Conclusions:**

This study demonstrates, for the first time, that both hbTESS models present similar *in vitro* biological characteristics. However, mechanical properties differ and future *in vivo* experiments will aim to compare their wound healing potential.

Highlights

- This study describes for the first time a side-by-side comparison of two clinical human bilayered tissue-engineered skin substitutes (human plasma-based and self-assembled skin substitutes).
- Using the same human skin cells for their manufacture, the self-assembly approach provided better *in vitro* mechanical properties although human plasma-based skin substitutes were produced faster.
- Similar *in vitro* biological characteristics were observed for both hbTESS models. However, future comparative clinical studies will aim to achieve a complete characterization of their *in vivo* wound healing potential.

## Background

Split-thickness skin autograft is the gold standard treatment for burns requiring grafts. However, the lack of donor tissue [1] and delayed intervention can lead to hypothermia, sepsis, chronic wounds and even death [2]. Human tissue-engineered skin substitutes (hTESSs) are a promising advanced therapy for the treatment of split- or full-thickness skin injuries. In the search for personalized medicine, different cell compositions, dermal matrix biomaterials or manufacturing processes are under evaluation to produce hTESSs [3].

hTESS cell composition has changed from cultured epithelial (keratinocytes only) [4–6] or dermal (fibroblasts only) [7–9] substitutes, to bilayered (keratinocytes and fibroblasts) [1, 10–16] substitutes, to get closer to native skin properties. Despite the lack of important structures such as blood vessels, nerves, hair follicles, sweat and sebaceous glands, these bilayered substitutes are a valuable solution as permanent coverage of full-thickness wounds [17].

To produce human bilayered TESSs (hbTESSs) different biomaterials have been proposed as dermal matrices [3]. Among them, human plasma is used at Unidad de Producción Celular e Ingeniería Tisular (Spain) for manufacturing clinical biomaterial-based hbTESSs, referred to as human plasma-based skin substitutes (HPSSs). Briefly, cultured fibroblasts are first embedded into a human plasma-based hydrogel, which is mainly formed due to the presence of the clotting protein fibrin, and then keratinocytes are seeded and cultured on top [12, 14, 15]. This model has already achieved positive results when applied on severely burned patients, in terms of safety and skin barrier restoration [11, 12, 14]. Using plasma is advantageous because it contains growth factors and extracellular matrix (ECM) components that are involved in the first stages of the wound healing process and promote the proliferation of keratinocytes [18]. In addition, human plasma is easy to obtain by centrifugation from blood samples and production of plasma-based scaffolds as dermal substitutes is quick due to its rapid coagulation. Moreover, its combination with other biomaterials such as hyaluronic acid enhances angiogenesis and wound healing potential *in vivo* [19].

Scaffold-free approaches can also be used for skin substitutes’ production, such as the so-called self-assembly method, developed at the LOEX Tissue Engineering Laboratory (Université Laval, Canada) [1]. Using this method, dermal substitutes are generated without exogenous biomaterials, by autologous fibroblasts cultured with ascorbic acid over several weeks post-confluency to induce the production of their own ECM. Self-assembled dermal substitutes support keratinocytes’ growth and differentiation into multilayer epidermis [20–22], generating functional hbTESSs, referred to as self-assembled skin substitutes (SASSs). SASSs have been successfully used for the treatment of patients with severe burns, with good engraftment rates and low scarring events [1, 23, 24].

Depending on the manufacturing method, hbTESSs’ properties are expected to vary in terms of mechanical and biological characteristics. Inter-individual variability regarding the cell’s behavior makes comparison between these models difficult. A direct comparison using the same cells under similar experimental conditions could provide useful data in order to choose the best personalized treatment for each patient.

**Figure 1:**
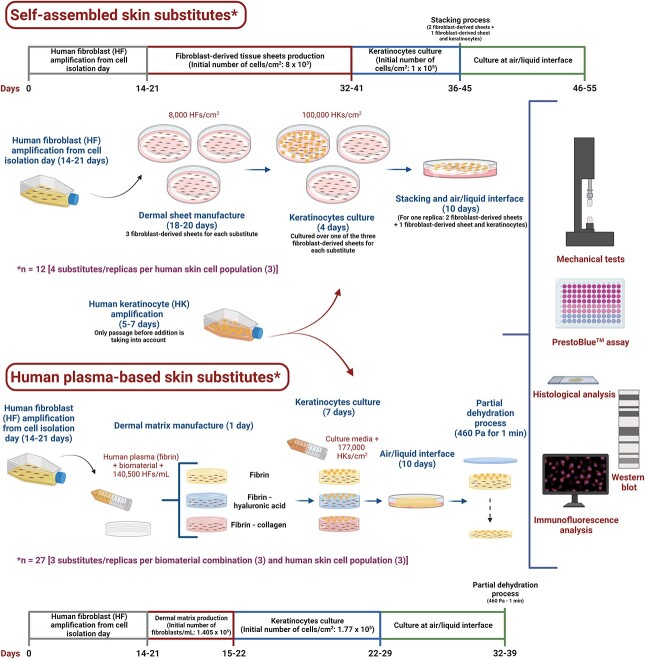
Graphical abstract and schematic representation of both manufacturing processes. Created with BioRender.com

Hence, a collaboration between the LOEX Tissue Engineering Laboratory (Canada) and the Unidad de Producción Celular e Ingeniería Tisular (Spain) was undertaken to compare the histological structure, mechanical and biological properties of i) SASSs and ii) three variations of the HPSS model [a) human plasma as unique biomaterial (Fibrin), b) human plasma combined with hyaluronic acid (Fibrin-HA) and c) human plasma combined with collagen (Fibrin-Col)] produced side-by-side with the same cell populations and under similar conditions (Figure 1).

## Methods

### Tissue and cell culture

Human keratinocytes and fibroblasts were isolated, as previously described [21, 25, 26], from different anatomical regions (chest [n = 2] and abdomen [n = 1]) of three human adult skin biopsies from healthy patients (22, 49 and 55 years old). Dermal fibroblasts were cultured in fibroblast medium (Dulbecco-Vogt modified Eagle medium [Gibco™, Waltham, MA] supplemented with 10% Avantor Seradigm FB Essence serum [Avantor®, Radnor, PA], 100 U/mL penicillin [Sigma-Aldrich, St. Louis, MO] and 25 μg/mL gentamicin [Gemini Bio, West Sacramento, CA]). Keratinocytes were grown on a *feeder* layer of irradiated human fibroblasts and cultured in keratinocyte medium (Dulbecco-Vogt modified Eagle medium [Gibco™]: Ham’s F12 [Gibco™], ratio 3:1, 24.25 μg/mL adenine [Sigma-Aldrich], 5 μg/mL insulin [Sigma-Aldrich], 0.4 μg/mL hydrocortisone [Galenova, Saint-Hyacinthe, Canada], 0.212 μg/mL isoproterenol hydrochloride [Sigma-Aldrich], 5% bovine HyClone FetalClone II serum [GE Healthcare, Chicago, IL], 10 ng/mL human epidermal growth factor [Austral Biologicals, San Ramon, CA], 100 U/mL penicillin [Sigma-Aldrich] and 25 μg/mL gentamicin [Gemini Bio]). For hbTESS production, cells were used at passage one to three for fibroblasts and three to four for keratinocytes. Each skin cell population (keratinocytes and fibroblasts from the same donor) was used to produce several replicas of both hbTESS models (four replicas for the SASS model and three replicas for each biomaterial combination of the HPSS model).

### Human bilayered tissue engineered-skin substitute production

#### Self-assembled skin substitutes

SASSs were manufactured as previously described [21, 22, 27]. To produce one fibroblast-derived tissue sheet (three sheets were required for each SASS), fibroblasts were seeded at 8$\times$10^3^ cells/cm^2^ on 60$\times$15 mm Tissue Culture Dishes (Falcon—BD, Franklin Lakes, NJ) and cultured in fibroblast medium supplemented with 50 μg/mL ascorbic acid (Sigma-Aldrich) for 18–20 days. Then, keratinocytes were seeded over one of the three fibroblast-derived sheets at 1$\times$10^5^ cells/cm^2^ (for each SASS) and cultured for 4 days in keratinocyte medium containing 50 μg/mL ascorbic acid. Next, two fibroblast-derived sheets and one with keratinocytes on top (for each SASS) were stacked and cultured at the air-liquid interface. The resulting constructs were cultured for 10 days in keratinocyte medium exempt of epidermal growth factor and supplemented with 50 μg/mL ascorbic acid. Finally, SASSs were collected for analysis. For each skin cell population (keratinocytes and fibroblasts isolated from the same donor), four SASSs were manufactured (n = 12 [3 skin cell populations $\times$ 4 replicas]).

#### Human plasma-based skin substitutes

HPSSs were manufactured in 60$\times$15 mm Petri dishes (Fisherbrand™, Waltham, MA) as previously described [19]. Three different human plasma-based hydrogels (final volume: 10 mL) were prepared: plasma as unique biomaterial (Fibrin), combined with 0.75 mg/mL hyaluronic acid (Sigma-Aldrich; Fibrin-HA) or with 0.78 mg/mL collagen peptides (Biogelx Ltd, Lanarkshire, UK; Fibrin-Col). Fibroblasts at 1.405$\times$10^5^ cells/mL were embedded into hydrogels and cultured for 1 day in fibroblast medium supplemented with 50 μg/mL ascorbic acid. Next day, 1.77$\times$10^5^ keratinocytes/cm^2^ were seeded on top and cultured for 7 days in keratinocyte medium containing 50 μg/mL of ascorbic acid. Then, HPSSs were cultured at the air-liquid interface for 10 days in keratinocyte medium exempt of epidermal growth factor and supplemented with 50 μg/mL ascorbic acid. Before analyzing, to enhance the biomechanical properties [28], hbTESSs were partially dehydrated under pressure using a glass disc (460 Pa, for 1 minute). For each skin cell population (keratinocytes and fibroblasts isolated from the same donor) and biomaterial used, three HPSSs were manufactured (n = 27 [3 skin cell populations $\times$ 3 biomaterials $\times$ 3 replicas]).

Schematic representation of the timeline for both manufacturing processes is shown in Figure 1.

### Mechanical test analysis

#### Adhesive strength of the dermal-epidermal junction (peel test)

To assess the adhesive strength of the dermal-epidermal junction of skin substitutes, a peel test was performed as previously described by Larose, EA. *et al*. [29]. Briefly, a 10 mm wide strip was cut out of each sample. The dermis and the epidermis were then manually separated on a small part of the strip and were fixed in the grips of an Instron ElectroPuls E1000 (Instron Corporation, Norwood, MA) mechanical tester with a Dynacell® 10 Newton (N) load cell (accuracy of ±0.0005 N). The dermis was then pulled apart from the epidermis at a constant rate of 0.7 mm/s by the mechanical tester, while the loadcell recorded the force required to do so. Force-displacement data were plotted using MATLAB script (MathWorks, Natick, MA). Average adhesive strength was calculated using the data in the permanent regime section of the curve and normalized by the width of the strip. To confirm that peeling occurred at the dermal-epidermal junction, the test was interrupted before the complete separation of the two layers and a biopsy of the separation line was fixed and embedded for histology analysis by Masson’s Trichrome staining.

#### Tensile strength and elasticity

A dog-bone shaped specimen was cut out from each sample using a homemade metallic punch. Each sample was then mounted into an Instron ElectroPuls E1000 mechanical tester (Instron Corporation) with a Dynacell® 10 Newton (N) load cell (accuracy of ±0.0005 N) and stretched at a constant 0.2 mm/s rate until complete failure. The distance between the two grips was considered as the gauge length, and the cross-section area was determined by multiplying the width of the punch (3 mm) and the thickness of the sample measured on the histological slides. The stress–strain curves were plotted using a MATLAB script (MathWorks) according to the recorded force-displacement data, sample cross-section area and gauge length. The elastic modulus (slope of the linear region) and ultimate tensile stress (highest stress value recorded before failure) were then determined using the plotted curve. The ultimate tensile strength (UTS) represents the maximal amount of stress that the hbTESS can support before failure. The elastic modulus represents the ability of the hbTESS to be stretched and to return to its original shape after unloading. A high elastic modulus represents a stiffer behavior.

### Histological analysis

For histological analysis, samples were fixed in formaldehyde 3.7% (pH = 7) and embedded in paraffin. Five-micrometer thick sections were stained with Hematoxylin and Eosin and Masson’s Trichrome (Weigert’s hematoxylin, fuchsin-ponceau and aniline blue). Digital images were acquired using an Axio Imager.Z2 microscope coupled with AxioCam ICc1 (brightfield) camera (Carl Zeiss Canada Ltd, North York, Canada). These samples were used for thickness measurements.

### Cell metabolic activity

Cell metabolic activity of hbTESSs was evaluated using PrestoBlue™ (Invitrogen™, Waltham, MA); a colorimetric assay which allows to measure resazurin reduction level (absorbance at 570 and 600 nm) by living cells. To that purpose, a sample of 10 mm diameter was taken from each hbTESS and incubated at 37°C in a PrestoBlue™-phosphate buffered saline (1/10) solution for 20 hours. Then the supernatant was collected and absorbance was measured using a Thermo Scientific Varioskan® Flash. The percentage reduction was calculated using the following equation:


$$\% Reduction=\frac{\left(O2\ x\ A1\right)-\left(O1\ x\ A2\right)}{\left(R1\ x\ C2\right)-\left(R2\ x\ C1\right)} x 100$$

– *O*1: molar extinction coefficient (E) of oxidized PrestoBlue™ (Blue) at 570 nm = > 80 586
– *O*2: E of oxidized PrestoBlue™ (Blue) at 600 nm = > 117 216
– *R*1: E of reduced PrestoBlue™ (Red) at 570 nm = > 155 677
– *R*2: E of reduced PrestoBlue™ (Red) at 600 nm = > 14 652
– *A*1: absorbance of test wells at 570 nm
– *A*2: absorbance of test wells at 600 nm
– *C*1: absorbance of control well (media plus PrestoBlue™ but no cells) at 570 nm
*C*2: absorbance of control well (media plus PrestoBlue™ but no cells) at 600 nm

### DNA quantification assay

The Kit DNeasy Blood & Tissue (Qiagen, Hilden, Germany) was used to extract DNA from frozen samples of each previously weighted hbTESS, and Quant-iT Picogreen dsDNA Assay Kit (Invitrogen) was used for DNA quantification by fluorescence (485 nm/520 nm) and measured using a Thermo Scientific Varioskan® Flash.

### Immunofluorescence analysis

For immunofluorescence analysis, the protocol has been previously described [30]. Briefly, samples were embedded in Tissue-Tek OCT compound (Sakura Finetek Inc., Torrance, CA) and frozen in liquid nitrogen. Indirect immunofluorescence assays were performed on 5 μm-thick cryosections permeabilized with acetone (10 min at −20°C). The antibodies used were: mouse monoclonal anti-human keratin (K) 10 (Abcam, Cambridge, UK; 1:500), rabbit polyclonal anti-human type IV collagen (Novus Biologicals, Toronto, Canada; 1:500), anti-human Ki67 (Abcam; 1:400), anti-human Loricrin (Biolegend, San Diego, CA; 1:1000), guinea polyclonal anti-human K15 (ARP, Waltham, MA; 1:1000). Negative controls which consisted in the omission of primary antibodies during the labeling reaction were included and normal human skin was used as a positive control. As secondary antibodies Alexa-488-conjugated donkey anti-rabbit (Life technologies, Waltham, MA; 1:1600), Alexa-594-conjugated donkey anti-mouse (Life technologies; 1:1600) and Alexa-594-conjugated goat anti-guinea pig (Life technologies; 1:200) were used. For K19 analysis, direct immunofluorescence assay was performed using mouse anti-human keratin 19-Cyanine 3 (clone A53-B/A244, gift from U. Karsten, Institute of Biological Sciences, University of Rostock, Germany; 1:200). Cell nuclei were counterstained with Hoechst reagent 33258 (Sigma-Aldrich).

Immunofluorescence sections were observed under a Zeiss Axio Imager.Z2 microscope (Carl Zeiss Canada Ltd) and photographed with Axiocam HRm Monochrome digital camera. Images were processed using AxioVision 4.8.2 software (Carl Zeiss Canada Ltd).

#### Percentage of positive basal epidermal cells analysis

ImageJ software (NIH, Bethesda, MD) was used to process immunofluorescence pictures. For each intra-replicate, three different pictures of the marked protein of interest and the same three pictures of Hoechst were chosen. After erasing dermal cells counterstained with Hoechst, positive basal epidermal cells for the protein of interest and all epidermal cells in Hoechst were counted. Finally, the percentage of positive basal epidermal cells by total number of epidermal cells was calculated for each picture and the mean value for each intra-replicate was calculated and used for statistical analysis.

### Western blot analysis

After the culture process, small samples of hbTESSs were collected and directly frozen in liquid nitrogen. The samples were then ground using a CryoMill (Retsch, Haan, Germany) with two steel beads (Ø = 5 mm) per sample and six shaking cycles of 1 minute at 24 Hz. Cell lysis was achieved using a PBS 1X solution containing 1% NP40 (Bio Basic, Amherst, NY), 1% Triton-X-100 (Bio-Rad, Hercules, CA), 0.5% sodium deoxycholate (Fisher Chemical, Waltham, MA), 0.1% SDS (Bio-Rad) and 1% protease/phosphatase inhibitor (Cell Signaling, Danvers, MA). Supernatants were collected after centrifugation at 13000 g for 10 minutes at 4°C. The total protein content was evaluated with a Micro BCA™ Protein Assay Kit (Thermo Scientific, Waltham, MA).

For each sample, 20 μg of proteins were loaded on 10% SDS-acrylamide gels (Bio-Rad). After 3 hours of migration at 80 V, proteins were electro-transferred for 1.5 hours at 4°C on Nitrocellulose Blotting Membrane (Amersham™ Protran™, Sigma-Aldrich). Membranes were blocked for 30 minutes at room temperature (RT) in TBS 1X containing 5% skimmed milk (Bio Basic) and 0.5% Tween 20 (Sigma-Aldrich), and then incubated overnight (ON) at 4°C with primary antibodies: rabbit polyclonal anti-human type I and IV collagen (Novus Biologicals; 1:1500 and 1:4000, respectively) and mouse monoclonal anti-human β-Actin (Sigma-Aldrich; 1:5000–10 000) as control. Membranes were washed eight times and incubated over agitation for 1 hour at RT with horseradish peroxidase-conjugated goat anti-rabbit and anti-mouse (Invitrogen; 1:5000). To reveal antibody-binding sites, SuperSignal™ West Dura (Thermo Scientific) was used. The signal was detected using a Fusion Fx7 (Vilber Lourmat, Collégien, France) and analyzed with ImageJ software (NIH, Bethesda, MD).

### Transmission electron microscopy (TEM)

Skin substitutes were fixed with 2.5% glutaraldehyde (Electron Microscopy Sciences, Hatfield, PA) in 0.1 M cacodylate buffer (Mecalab, Montreal, Canada) at 4°C ON. The next day, the fixative was removed and samples were washed four times with cacodylate buffer at 4°C for 15 minutes. Finally, samples were kept in cacodylate solution and sent to Microscopy Service of Université Laval for inclusion and staining with uranyl acetate and lead citrate. Images were taken with a LIBRA 120 PLUS Transmission Electron Microscope (Carl Zeiss SMT, Oberkochen, Germany) at Scientific Instrumentation Center of Universidad de Granada.

### Statistical analysis

Microsoft Excel software was used for primary analysis of raw data, and GraphPad Prism 8 (San Diego, CA) was used for statistical analysis and graphs. Data for each group of analysis and parameter obeyed a normal distribution, corroborated by Shapiro–Wilk test. Then, ordinary one-way ANOVA and Tukey’s multiple comparison test were used to analyze the results of each assay. All values are presented as mean value ± standard deviation (SD). The significance threshold was set at 0.05.

### Ethics

This study was conducted according to the Declaration of Helsinki and was approved by the research ethics committee for human subjects of the CHU de Quebec-Université Laval. All tissue donors provided informed written consent.

## Results

### Histological appearance and adhesive strength of the dermal-epidermal junction of hbTESSs

**Figure 2:**
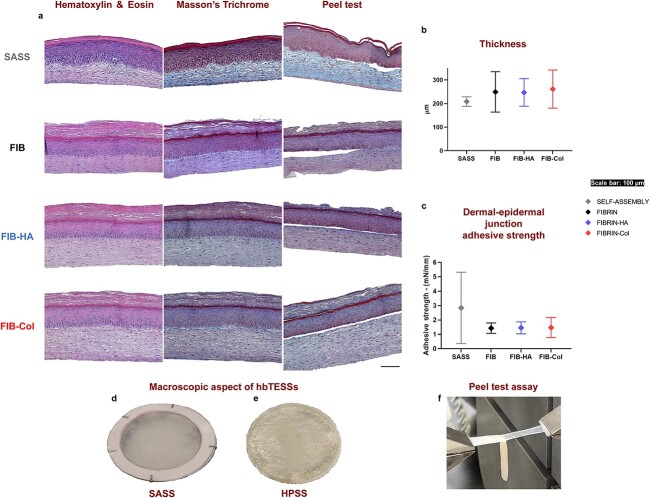
Histological pictures, thickness measurement and dermal-epidermal junction adhesive strength of hbTESSs evaluated by the peel test. (**a**) From left to right in all groups: Hematoxylin/Eosin staining, Masson’s Trichrome staining and confirmation that peeling occurred at the adequate localization, the dermal-epidermal junction. (**b**) Measure of the hbTESS thickness using histological pictures (N = 3; n = 12, 9, 9, 9 for each group, respectively). (**c**) Dermal-epidermal junction adhesive strength evaluation (N = 3; n = 18, 10, 10, 9 for each group, respectively). Macroscopic aspect of (**d**) SASS and (**e**) HPSS. (**f**) Representative picture of peel test assay. Scale bar: 100 μm. One-way ANOVA and Tukey’s multiple comparison tests. Data is shown as mean value ± SD. *hbTESSs* human bilayered tissue-engineered skin substitutes, *SASS* self-assembled skin substitute, *FIB/FIBRIN* hbTESS constituted of human plasma as biomaterial, *FIB-HA/FIBRIN-HA* hbTESS constituted of human plasma and hyaluronic acid as biomaterials, *FIB-Col/FIBRIN-Col* hbTESS constituted of human plasma and collagen as biomaterials, *HPSS* human plasma-based skin substitute

Human healthy skin has a complex structure and a large amount of collagen is found in the dermis (stained in blue by Masson’s trichrome staining; Figure S1, see online supplementary material). Regarding skin substitutes, despite differing manufacturing times [46–55 days for SASSs and 32–39 days for HPSSs (Figure 1)], all hbTESSs developed a differentiated epidermis with a granular layer and a cornified envelope as shown by histology (Figure 2). However, HPSSs exhibited more accumulation of stratum corneum (Figure 2a) and were slightly thicker than SASSs (Figure 2b). Moreover, Masson’s trichrome staining (Figure 2a) indicated that collagen was more homogeneously distributed through the dermal layer in SASSs (Figure 2a) compared with HPSSs. The staining of the ECM under the basement membrane was bluer in the SASSs, while it appeared purplish in the rest of the dermal layers (Figure 2a).

Therefore, histological observation of skin substitute models showed a proper bilayered structure without detachment or separation between dermis and epidermis (Figure 2; Hematoxylin/Eosin and Masson’s Trichrome staining). To characterize the adhesive strength of the dermal-epidermal junction (DEJ), peel tests [29] were performed. The adhesive strengths of the DEJ for the Fibrin, Fibrin-HA and Fibrin-Col skin substitutes were similar, with a mean adhesive strength of 1.47 ± 0.47 mN/mm, which was 1.93 times lower compared to SASSs with a mean adhesive strength of 2.84 ± 2.48 mN/mm (Figure 2c). Although experiments were repeated, data collected for SASSs were more variable depending on the cell donor used for manufacturing (Figure S2, see online supplementary material). Histology was used after peel testing to confirm that the detachment occurred at the DEJ (Figure 2a) indicating that the peel test measured the force anchoring the epidermis to the dermis.

### Tensile strength and elasticity of hbTESSs

**Figure 3:**
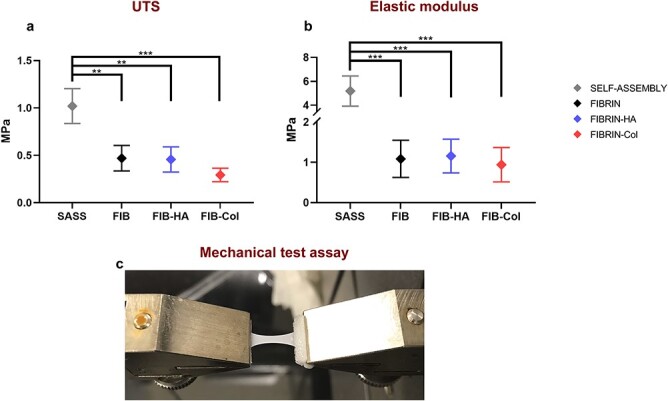
Mechanical properties’ analysis of the different hbTESSs (SASS, Fibrin, Fibrin-HA and Fibrin-Col). (**a**) Evaluation of UTS (N = 3; n = 12, 9, 9, 9 for each group, respectively). (**b**) Elastic modulus’ results (N = 3; n = 12, 9, 9, 9 for each group, respectively). (**c**) Representative picture of mechanical test assay. Statistical significance: ^*^^*^p < 0.01; ^*^^*^^*^p < 0.001, One-way ANOVA and Tukey’s multiple comparison tests. Data is shown as mean value ± SD. *hbTESSs* human bilayered tissue-engineered skin substitutes, *SASS* self-assembled skin substitute, *FIB/FIBRIN* hbTESS constituted of human plasma as biomaterial, *FIB-HA/FIBRIN-HA* hbTESS constituted of human plasma and hyaluronic acid as biomaterials, *FIB-Col/FIBRIN-Col* hbTESS constituted of human plasma and collagen as biomaterials, *UTS* ultimate tensile strength

Mechanical analysis was completed by the evaluation of tensile strength and elastic properties of hbTESSs (Figure 3). SASSs presented higher ultimate tensile strength (UTS) values than HPSSs. Therefore, SASSs were more resistant to tensile forces (*p-*value < 0.01). Surprisingly, considering the properties of native proteins used as biomaterials, Fibrin-Col was less resistant compared to SASSs (*p-*value < 0.001) (Figure 3a).

On the other hand, the elastic modulus indicated that no significant differences were observed between the three human plasma-based skin substitutes. However, SASSs were significantly stiffer than the other hbTESSs (*p-*value < 0.001) (Figure 3b).

### Cell metabolic activity and DNA quantification of hbTESSs

**Figure 4:**
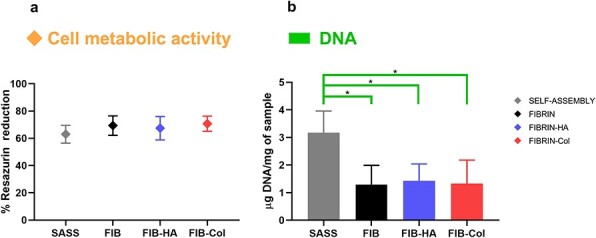
Cell metabolic activity and amount of DNA in hbTESSs (SASS, Fibrin, Fibrin-HA and Fibrin-Col). (**a**) Cell metabolic activity measured by Presto Blue™ assay and (**b**) DNA quantification by mg of tissue (N = 3; n = 18, 10, 10, 10 for each group, respectively). Statistical significance: ^*^p < 0.05, One-way ANOVA and Tukey’s multiple comparison tests. Data is shown as mean value ± SD. *hbTESSs* human bilayered tissue-engineered skin substitutes, *SASS* self-assembled skin substitute, *FIB/FIBRIN* hbTESS constituted of human plasma as biomaterial, *FIB-HA/FIBRIN-HA* hbTESS constituted of human plasma and hyaluronic acid as biomaterials, *FIB-Col/FIBRIN-Col* hbTESS constituted of human plasma and collagen as biomaterials

At the end of the culture process, cell metabolic activity measured by Presto Blue™ assay and amount of DNA per mg of sample were evaluated (Figure 4). Considering the percentage of resazurin reduction, cell metabolic activity was slightly lower in SASSs although no significant differences were observed compared to HPSSs (Figure 4a). In contrast, the amount of DNA per mg of sample was significantly higher in SASSs compared to HPSSs (Figure 4b; *p-*value < 0.05), suggesting that the number of cells in human plasma-based skin substitutes was lower.

### Immunofluorescence analysis of hbTESSs

**Figure 5:**
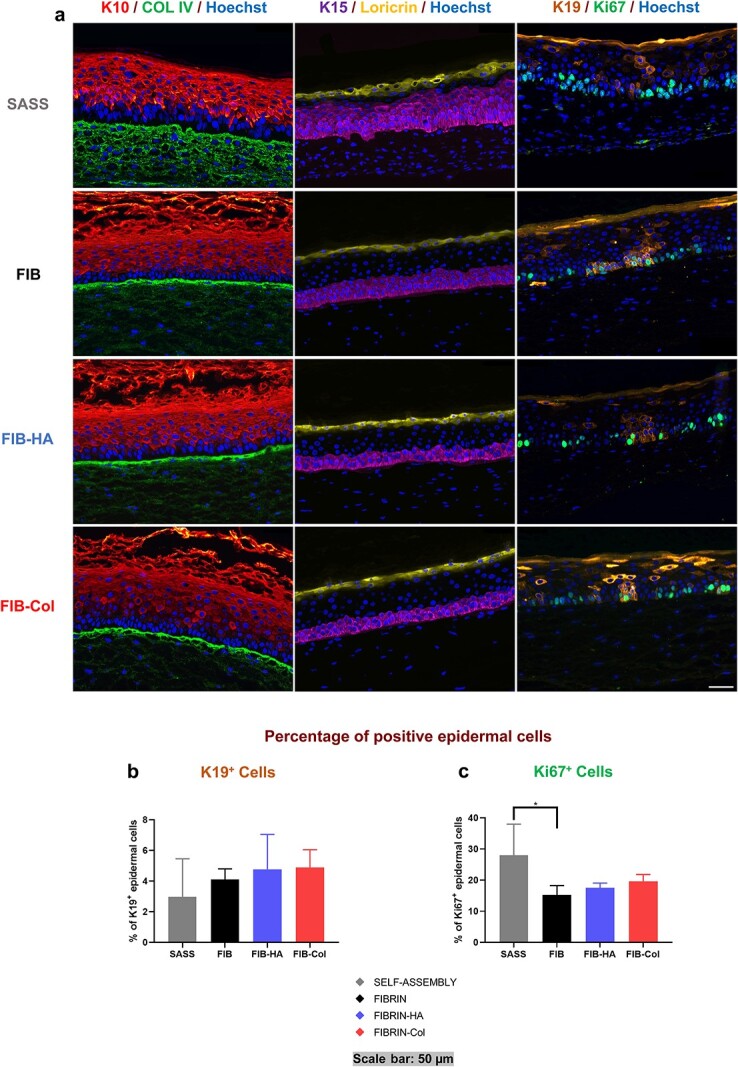
Immunofluorescence pictures and analysis of hbTESSs. (**a**) From left to right in all groups: I) K10 (red)/COL IV (green)/Hoechst nucleus (blue), II) K15 (purple)/Loricrin (yellow)/Hoechst nucleus (blue) and III) K19 (orange)/Ki67 (green)/Hoechst nucleus (blue) staining. (**b**) Percentage of epidermal cells that are K19^+^ and localized in the basal layer (N = 3; n = 16, 9, 9, 9 for each group, respectively). (**c**) Percentage of epidermal cells that are Ki67^+^ and localized in the basal layer (N = 3; n = 16, 9, 9, 9 for each group, respectively). Scale bar: 50 μm. Statistical significance: ^*^p < 0.05, One-way ANOVA and Tukey’s multiple comparison tests. Data is shown as mean value ± SD. *hbTESSs* human bilayered tissue-engineered skin substitutes, *K10* Keratin 10, *COL IV* Type IV collagen, *K15* Keratin 15, *K19* Keratin 19, *SASS* self-assembled skin substitute, *FIB/FIBRIN* hbTESS constituted of human plasma as biomaterial, *FIB-HA/FIBRIN-HA* hbTESS constituted of human plasma and hyaluronic acid as biomaterials, *FIB-Col/FIBRIN-Col* hbTESS constituted of human plasma and collagen as biomaterials

To achieve the characterization of both hbTESS models, different skin associated markers were studied by double immunofluorescence labeling. For Keratin 19 (K19) and Ki67 markers, the percentage of positive basal epidermal cells by total number of epidermal cells was measured to provide quantitative results (Figure 5).

#### Keratin 10/Type IV collagen

K10 in healthy human skin is found across suprabasal layers (Figure S1, see online supplementary material). Both hbTESS models expressed K10 through the keratinocytes of suprabasal epidermal layers (Figure 5a; Red). According to the histological results, HPSSs presented a less compact stratum corneum positive for K10 (Figure 5a). Type IV collagen was expressed at the DEJ in all tested hbTESSs (Figure 5a; Green), like in native human skin control (Figure S1, see online supplementary material), but a diffuse expression was observed in the dermis of SASSs (Figure 5a) whereas it was mainly located at the DEJ in HPSSs (Figure 5a).

#### Keratin 15/Loricrin

K15 expression (Figure 5a; Purple) was not confined to the basal layer like in native human skin (Figure S1, see online supplementary material) but also to upper cell layers in all tested hbTESSs, and it was more pronounced in SASSs (Figure 5a). Loricrin expression, like in the granular layer of the native human epidermis (Figure S1, see online supplementary material), was similar between all tested hbTESSs (Figure 5a; Yellow).

#### Keratin 19/Ki67

K19 is a stem-cell-associated marker and Ki67, a proliferative marker, present in a small proportion of epidermal cells (0.51 ± 0.37% for K19 and 12.66 ± 2.75% for Ki67) and expressed in cells of the basal layer that are in contact with the basement membrane (Figure S1, see online supplementary material). K19^+^ (Orange) and Ki67^+^ (Green) cells were observed in the basal layer of epidermis of all tested hbTESSs (Figure 5a). Notably, a K19 non-specific staining was also visualized in the suprabasal layers but was not considered for analysis.

A tendency toward a slightly higher proportion of epidermal cells expressing K19 was observed in HPSSs compared to SASSs, although no significant differences were measured (Figure 5b). In contrast, the percentage of epidermal cells expressing Ki67 was higher in SASSs, although a significant difference was only observed compared to Fibrin (*p-*value < 0.05) (Figure 5c).

### Type I and type IV collagen protein quantification in hbTESSs

**Figure 6:**
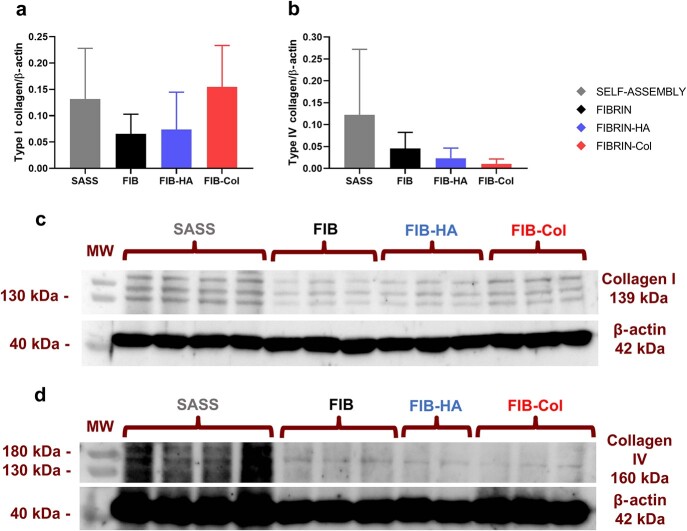
Western blot analysis of type I and IV collagen of hbTESSs (SASS, Fibrin, Fibrin-HA and Fibrin-Col). (**a**) Ratio of type I collagen on β-actin protein (N = 3; n = 12, 9, 9, 9 for each group, respectively); (**b**) Ratio of type IV collagen on β-actin protein (N = 3; n = 12, 9, 9, 9 for each group, respectively); (**c**) and (**d**) Representative pictures of a type I (**c**) and a type IV (**d**) collagen Western blot. One-way ANOVA and Tukey’s multiple comparison tests. Data is shown as mean value ± SD. *hbTESSs* human bilayered tissue-engineered skin substitutes, *SASS* self-assembled skin substitute, *FIB/FIBRIN* hbTESS constituted of human plasma as biomaterial, *FIB-HA/FIBRIN-HA* hbTESS constituted of human plasma and hyaluronic acid as biomaterials, *FIB-Col/FIBRIN-Col* hbTESS constituted of human plasma and collagen as biomaterials *MW* molecular weight, *kDa* Kilodalton

Western blot quantification revealed that type I collagen was 2.20 times higher in Fibrin-Col skin substitutes and 1.89 times higher in SASSs compared to Fibrin and Fibrin-HA skin substitutes although no significant differences were observed (Figure 6a).

Quantification of type IV collagen by Western blot revealed that, although without significance, more protein was present in SASS; 2.65 times higher than Fibrin, 5.3 times higher than Fibrin-HA and 11.1 times higher than Fibrin-Col skin substitutes (Figure 6b), a result consistent with the immunofluorescence tissue labeling.

Representative pictures of both, type I and type IV collagen Western blots, were included into Figure 6c and 6d, respectively.

### Ultrastructural aspect of human plasma-based skin substitutes

**Figure 7:**
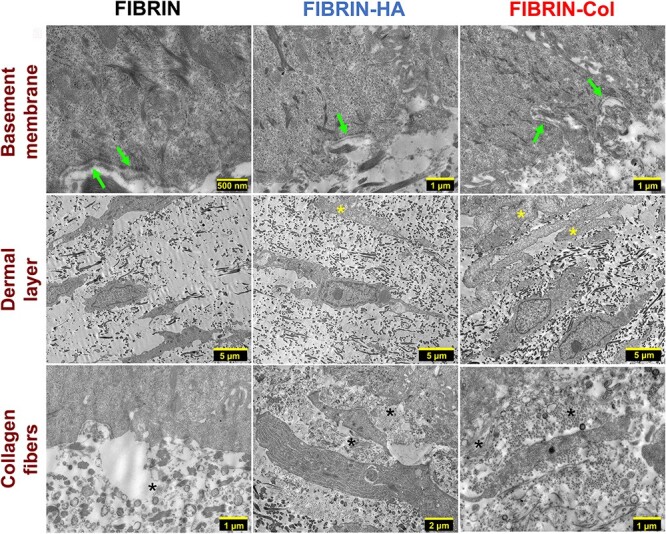
Transmission electron microscopy (TEM) pictures of human plasma-based skin substitutes (HPSSs). Basement membrane pictures (arrows) of Fibrin, Fibrin-HA and Fibrin-Col skin substitutes. Dermal layer ultrastructure of Fibrin, Fibrin-HA and Fibrin-Col skin substitutes; asterisks indicate the presence of biomaterial deposits. Collagen fibers appearance of Fibrin, Fibrin-HA and Fibrin-Col skin substitutes; asterisks indicate the presence and orientation of collagen fiber packages. *hbTESSs* human bilayered tissue-engineered skin substitutes, *FIB/FIBRIN *hbTESS constituted of human plasma as biomaterial, *FIB-HA/FIBRIN-HA* hbTESS constituted of human plasma and hyaluronic acid as biomaterials, *FIB-Col/FIBRIN-Col* hbTESS constituted of human plasma andcollagen as biomaterials

The ultrastructure of SASSs has been previously characterized by Transmission Electron Microscopy (TEM), demonstrating the presence of a basement membrane, with numerous hemidesmosomes, as well as fibroblasts surrounded by the ECM they produced, comprising collagen fibers in the dermal layer after culturing at the air-liquid interface [1, 20, 22, 29]. To define the ultrastructural aspect of human plasma-based skin substitutes, TEM analysis was conducted (Figure 7). A discontinuous basement membrane without hemidesmosomes was visualized at the DEJ (Figure 7), indicating that the basement membrane assembly was initiated. Fibroblasts and ECM elements were also observed in all HPSSs (Figure 7), including collagen fibers (Figure 7), whose relative amounts were consistent with the type I collagen quantified by Western blot (Figure 6).

## Discussion

In this study, we compared two hbTESS models already applied for the treatment of severely burned patients [1, 14, 16]. Although the safety of these models has already been proven in a clinical setting, direct comparison using the same cell populations has provided useful *in vitro* data on their respective mechanical and biological properties. Moreover, the addition of hyaluronic acid or collagen to the human plasma-based skin substitutes revealed only minor effects on hbTESSs properties.

One main difference between both models is the culture time. In this study, SASSs were generated in 46–55 days, which is similar to the delay previously reported before patients get their first grafts using SASSs [1], whereas HPSSs were manufactured in 32–39 days. This manufacturing time could be longer depending on the patient, for example, due to his/her age, a factor that could influence cell proliferation [31]. Moreover, some studies have reported that infections and septic complications are the main cause of death in severely burned patients [32, 33]. Since the first protective barrier and several adnexal structures are damaged the human body is highly exposed, therefore, a short hbTESS production time is a significant asset. Unfortunately, both manufacturing processes are still long and an immediate grafting of autologous tissue-engineered skin substitutes is not possible. However, the benefits of using autologous therapies when autografts cannot be applied are higher, and other clinical strategies to diminish the risk of infection while waiting for a definitive treatment to replace the epidermis, should be developed. For example, Novosorb™ Biodegradable Temporizing Matrix was applied early on burn wounds until SASS was available for grafting with good results in one patient [23]. Nevertheless, other considerations apart from manufacturing time are equally important such as a high graft take, prevention of the development of hypertrophic scars, reduction in the number of additional surgeries (for SASSs engraftment [1]) and long-term persistence of the grafted hbTESSs.

In terms of clinical use, human plasma-based skin substitutes need to be dehydrated in order to improve their biomechanical properties [28], but otherwise the grafting procedure is similar for both models; after surgical debridement, SASSs and HPSSs are applied to the wound bed and secured with staples or surgical glues (in SASSs [1]). For each model, post-operative treatment is the same for autografts and skin substitutes, but it differs in the two countries. Commercial gauzes were different; for SASSs, a conventional type of bolster is applied for 5–7 days [1], while for HPSSs, gauze changes and physiological saline solution and soap washes are applied every 2 days.

The study of both hbTESS models using the peel test methodology [29] revealed that the DEJ strength of SASSs was higher than in HPSSs. This lower adhesive force is consistent with TEM results; where basement membrane formation was not completed after 10 days of the culture at the air-liquid interface in contrast to SASSs where previous studies reported a continuous basement membrane using the same culture conditions [22, 29, 34]. Moreover, analysis of type IV collagen expression, the primary collagen found in basement membranes and a major component of the DEJ [35], demonstrated that it was overexpressed in the dermis of SASSs. The lower amount into the dermis of human plasma-based skin substitutes may be a matter of culture time and/or because of embedding the fibroblasts into the hydrogel [36]. The biomaterial could also diminish fibroblast interaction with type IV collagen, expressed by keratinocytes [37, 38], resulting in a lack of hemidesmosomes and basement membrane formation. The importance of the presence of a basement membrane is likely to prevent the delamination of the epidermis from the dermis of the hbTESSs during manipulation at the time of grafting and thereafter. This is one of the factors that could explain the high SASS engraftment (98%) on severely burned patients [1]. However, the basement membrane could be formed after grafting *in vivo* as shown for biomaterial (collagen or human plasma)-based skin substitutes [39–41] and high engraftment has also been observed at discharge for this model (80–85%). Therefore, *in vivo* trials will be necessary to compare their regenerative potential.

Regarding tensile and elastic properties, the compression applied in human plasma-based hbTESSs creates a partial dehydration and aligns collagen [42] or fibrin fibers [28], generating stiffer and denser skin substitutes than fully hydrated models, improving their handling and rheological properties [28, 42], although similar behavior has been observed regardless of biomaterial combination. However, SASSs are stiffer and more resistant to tensile forces which could be explained by the longer culture period of dermal layers and also by the self-assembled nature of the ECM; more similar to native human dermis [20].

Concerning biological aspects, the lower amount of DNA per mg of tissue quantified in biomaterial-based skin substitutes compared with SASSs indicated that a lower number of cells formed the HPSSs. This result was consistent with the slightly higher proportion of epidermal cells expressing Ki67 observed in SASSs, suggesting hyperproliferation. Despite the higher number of cells seeded (5.1$\times$10^6^  *vs* 2.6$\times$10^5^ cells) and the longer epidermal culture time in HPSS protocol (17 *vs* 14 days), the much longer culture time required for each of the three fibroblast-derived sheets manufactured in SASSs (18–20 *vs* 1 day) resulted in more cells per mg of tissue. Moreover, although more physiologically similar in terms of structure than 2D cultures, the embedding of fibroblasts into a 3D hydrogel for human plasma-based skin substitutes may constrain cell growth [36] and explain the lower amount of DNA. In contrast, cell metabolic activity was higher in HPSSs which could be explained by the presence of human plasma factors such as platelet-derived growth factor-BB (PDGF-BB) or hepatocyte growth factor (HGF), known to increase epidermal cell metabolic activity [18, 43].

### Limitations

Three different human skin cell populations were used which could represent a limitation. However, the number of intra-replicas (between three and four) for each condition and the repetition of some experiments, using the same cell populations, confirmed the results, and allowed us to obtain accurate comparative outcomes.

## Conclusions

This *in vitro* study is the first side-by-side comparison of two clinical hbTESS models based on distinct manufacturing processes. Results reported that similar *in vitro* biological characteristics are observed, however, SASSs present better *in vitro* mechanical properties, although its manufacturing process is more time-consuming than for HPSSs. For patient treatment, evaluation of production time, biological characteristics, mechanical properties and the use of biomaterials or not are key points but not the only ones since graft take, long-term persistence and the number of additional surgeries required to treat scars are also important. Therefore, in addition to the safety of these two hbTESS models that has already been proven in patients, future comparative clinical trials (SASS *vs* HPSS) will provide the data required to achieve a complete characterization of their *in vivo* wound healing potential.

## Abbreviations

AU: Arbitrary Units; COL IV: Type IV collagen; DEJ: Dermal-epidermal junction; ECM: Extracellular Matrix; Fibrin-Col: Human plasma and collagen (biomaterial); Fibrin-HA: Human plasma and hyaluronic acid (biomaterial); Fibrin: Human plasma (biomaterial); hbTESS: Human bilayered tissue-engineered skin substitute; HGF: Hepatocyte growth factor; HPSS: Human plasma-based skin substitute; hTESS: Human tissue-engineered skin substitute; K: Keratin; PDGF-BB: Platelet-derived growth factor-BB; ON: Overnight; SASS: Self-assembled skin substitute; SD: Standard deviation; TEM: Transmission Electron Microscopy; UTS: Ultimate Tensile Strength

## Supplementary Material

Figure_S1_tkad043

Figure_S2_tkad043

## Data Availability

The authors declare that the data supporting the findings of this study are available within the paper and its supplementary information files.
