# Supplementary figures and images for: In vitro comparison of human plasma-based and self-assembled tissue-engineered skin substitutes: two different manufacturing processes for the treatment of deep and difficult to heal injuries
Source: Burns Trauma. 2023 Oct 31;11:tkad043. doi: 10.1093/burnst/tkad043 (PMC10615253; doi:10.1093/burnst/tkad043)

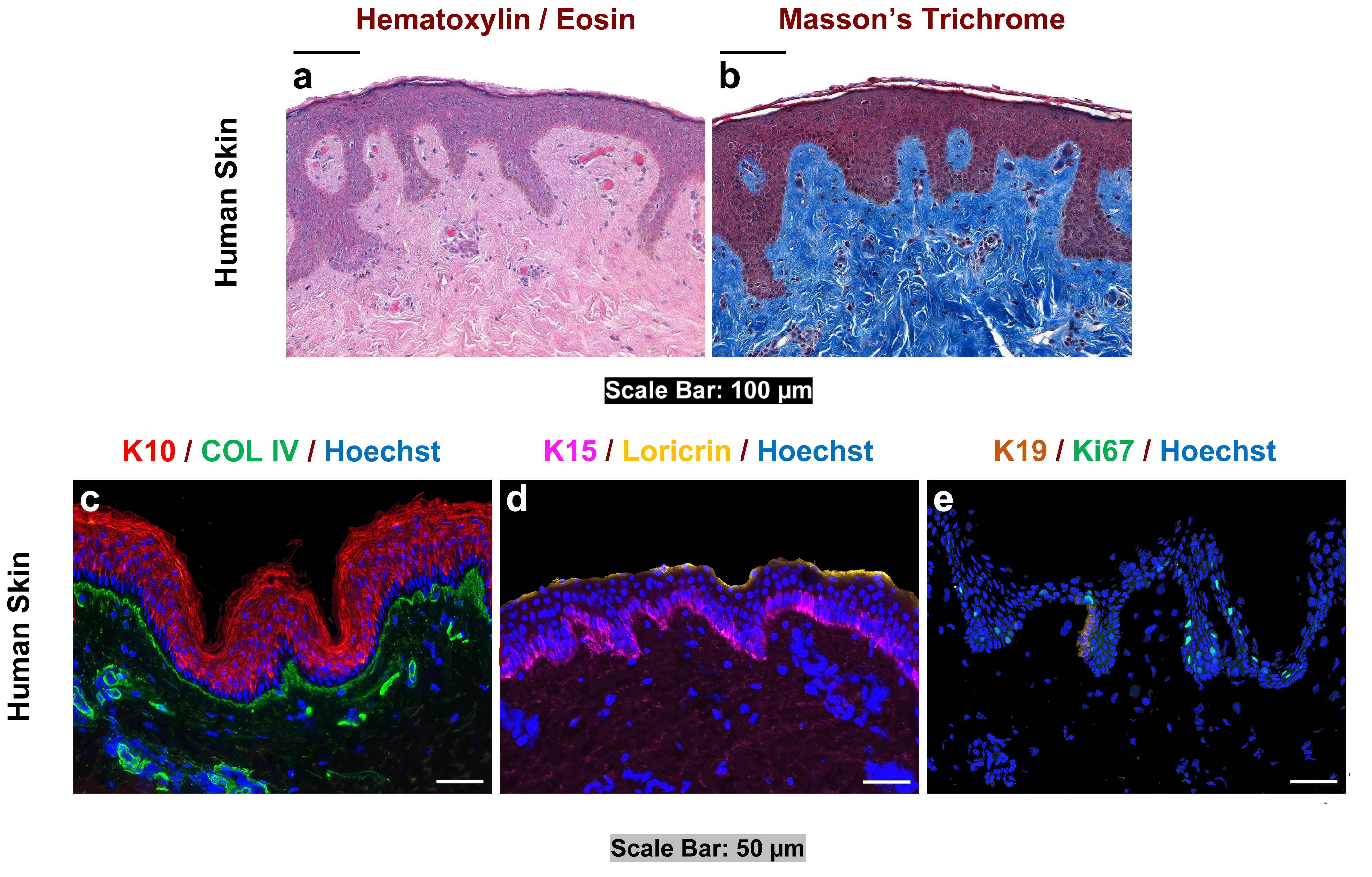

Supplement: Figure_S1_tkad043 [file figure_s1_tkad043.jpeg]

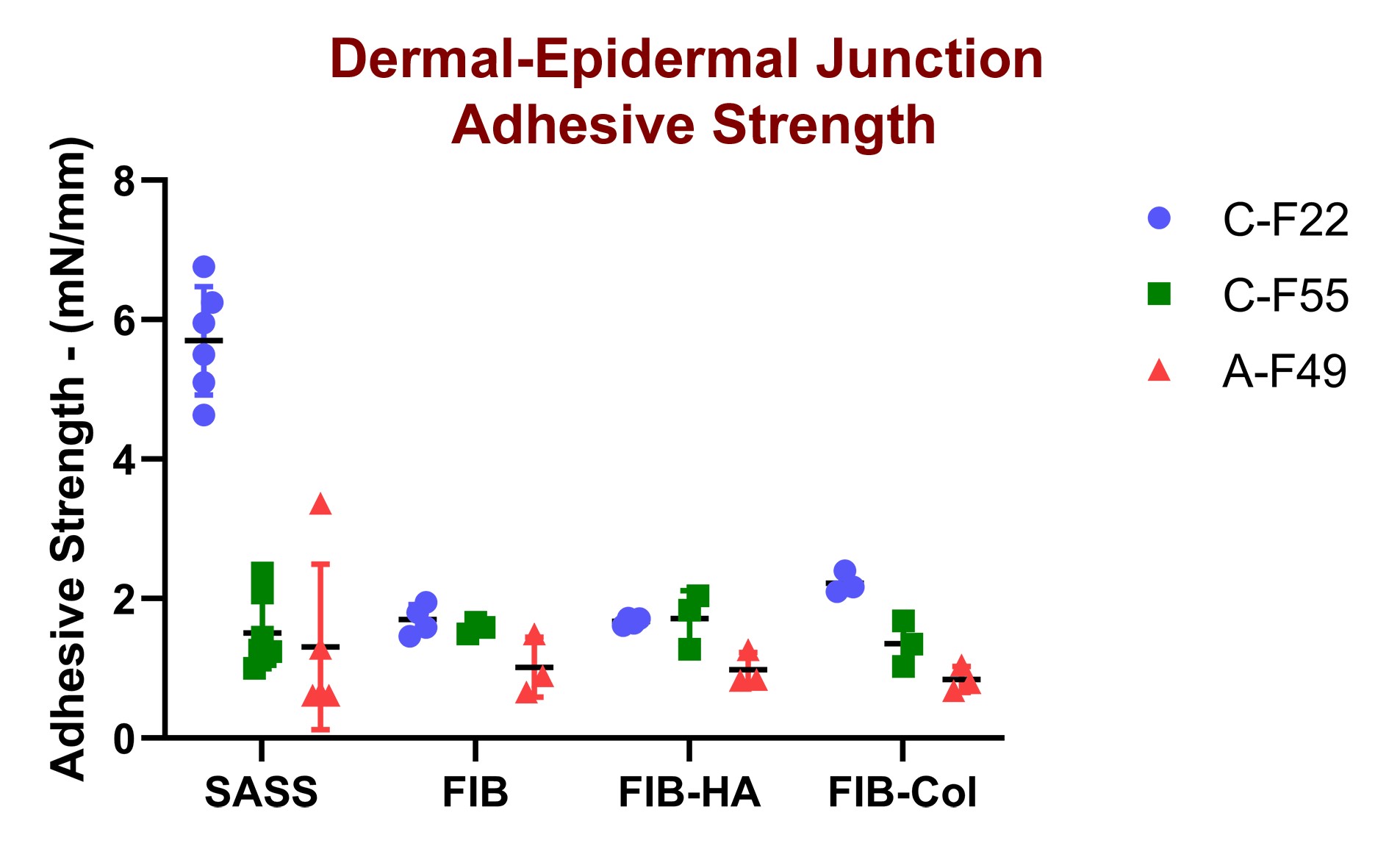

Supplement: Figure_S2_tkad043 [file figure_s2_tkad043.jpeg]
